# Editorial Note: Validation of commercial Mas receptor antibodies for utilization in Western Blotting, immunofluorescence and immunohistochemistry studies

**DOI:** 10.1371/journal.pone.0362513

**Published:** 2026-11-11

**Authors:** The PLOS One Editors

The *PLOS One* Editors issue this Editorial Note to inform readers that concerns have been raised about results presented in Fig 7 of this article [1] and Fig 1A of [2].
